# A Novel Cervical Spinal Cord Window Preparation Allows for Two-Photon Imaging of T-Cell Interactions with the Cervical Spinal Cord Microvasculature during Experimental Autoimmune Encephalomyelitis

**DOI:** 10.3389/fimmu.2017.00406

**Published:** 2017-04-11

**Authors:** Neda Haghayegh Jahromi, Heidi Tardent, Gaby Enzmann, Urban Deutsch, Naoto Kawakami, Stefan Bittner, Dietmar Vestweber, Frauke Zipp, Jens V. Stein, Britta Engelhardt

**Affiliations:** ^1^Theodor Kocher Institute, University of Bern, Bern, Switzerland; ^2^Max Planck Institute of Neurobiology, Martinsried, Germany; ^3^Institute of Clinical Neuroimmunology, Biomedical Center and University Hospital, Ludwig-Maximilians University of Munich, Martinsried, Germany; ^4^Focus Program Translational Neuroscience (FTN) and Immunotherapy (FZI), Rhine Main Neuroscience Network (rmn^2^), University Medical Center of the Johannes Gutenberg University, Mainz, Germany; ^5^Max-Planck-Institute of Molecular Biomedicine, Münster, Germany

**Keywords:** cervical spinal cord window, two-photon intravital microscopy, experimental autoimmune encephalomyelitis, blood–brain barrier, T-cell migration

## Abstract

T-cell migration across the blood–brain barrier (BBB) is a crucial step in the pathogenesis of experimental autoimmune encephalomyelitis (EAE), an animal model of multiple sclerosis (MS). Two-photon intravital microscopy (2P-IVM) has been established as a powerful tool to study cell–cell interactions in inflammatory EAE lesions in living animals. In EAE, central nervous system inflammation is strongly pronounced in the spinal cord, an organ in which 2P-IVM imaging is technically very challenging and has been limited to the lumbar spinal cord. Here, we describe a novel spinal cord window preparation allowing to use 2P-IVM to image immune cell interactions with the cervical spinal cord microvascular endothelium during EAE. We describe differences in the angioarchitecture of the cervical spinal cord versus the lumbar spinal cord, which will entail different hemodynamic parameters in these different vascular beds. Using T cells as an example, we demonstrate the suitability of this novel methodology in imaging the post-arrest multistep T-cell extravasation across the cervical spinal cord microvessels. The novel methodology includes an outlook to the analysis of the cellular pathway of T-cell diapedesis across the BBB by establishing visualization of endothelial junctions in this vascular bed.

## Introduction

Migration of autoaggressive T cells across the endothelial blood–brain barrier (BBB) is a critical step in the pathogenesis of experimental autoimmune encephalomyelitis (EAE), an animal model for multiple sclerosis (MS). EAE can be induced by adoptive transfer of *in vitro* activated neuroantigen specific CD4^+^ T cells into syngeneic susceptible recipients. These encephalitogenic CD4^+^ T cells have acquired the molecular keys allowing them to engage the cell adhesion and signaling molecules on the BBB allowing them to cross this barrier in a multistep process. Having crossed the BBB, these T cells become reactivated after recognition of their cognate antigen on antigen-presenting cells in the context of major histocompatibility class II (MHC class II) molecules and initiate an inflammatory cascade leading to inflammation, demyelination, and neurodegeneration (1–3).

Several research groups have employed real-time epifluorescence intravital microscopy (IVM) using a cranial window model to study the interaction of T cells within superficial brain microvessels during EAE. These studies demonstrated that P-selectin glycoprotein ligand-1 (PSGL-1) and α4-integrins are important for T-cell rolling in inflamed leptomeningeal brain vessels, while lymphocyte function associated antigen-1 and α4-integrins mediate T cell arrest (4, 5). These findings were confirmed by others who demonstrated that T-cell rolling and arrest in the superficial brain vessels exposed in the cranial window preparation are mediated by endothelial P-selectin and α4-integrins on T cells, respectively (6, 7).

We have pioneered preparation of a cervical spinal cord window in mice allowing to observe the interaction of activated encephalitogenic T cells with cervical spinal cord microvessels by real-time epifluorescence IVM (8). This study showed that during the initiation of EAE, interaction of encephalitogenic T cells with the spinal cord microvasculature is unique due to the predominant involvement of α4-integrins in capture and arrest of the T cells to the vascular wall and the lack of rolling (8).

In follow-up studies, we have adapted this cervical spinal cord window preparation to study T-cell interaction with spinal cord microvessels in mice suffering from EAE. One of these studies demonstrated that once neuroinflammation is established, T-cell interaction with the cervical spinal cord microvasculature is initiated by rolling. Interaction of PSGL-1 and its endothelial ligands E- and P-selectin was found to be essential for T-cell rolling in this vascular bed (9). In addition, this methodology has allowed to demonstrate that natalizumab, which is a humanized anti-α4 integrin antibody used for the treatment of relapsing-remitting MS, inhibits the firm adhesion but not the initial rolling or capture of human T cells within inflamed spinal cord microvessels during EAE in mice (10).

Real-time epifluorescence IVM is the preferred methodology to study rapid T-cell interactions with the vascular wall like T-cell rolling, which occurs at an average speed of 5–6 µm/s in superficial brain microvessels (4) and at about 18 µm/s in cervical spinal cord microvessels (9). Slower T-cell interactions with the BBB, e.g., post-arrest intravascular T-cell crawling at reported speeds of 5–15 μm/min or T-cell diapedesis across the BBB, which was reported to occur at minutes to hours after T-cell arrest (8, 11, 12) cannot be imaged by real-time epifluorescence IVM due to phototoxic effects. To this end, two-photon IVM (2P-IVM; 2PM) provides a powerful tool to image T-cell extravasation across the BBB over an extended period of time (12, 13).

A major bottleneck of *in vivo* 2P-IVM imaging is that it demands optical access to the central nervous system (CNS), which is covered by bone, the dura mater and the leptomeningeal layers (14). There are established protocols allowing for 2P-IVM of brain vessels by employing an open cranial window or a thinned skull preparation (4, 5). In addition, lumbar spinal cord windows have been described in rats and mice (15–17). To date, there is, however, no methodology available allowing to perform 2P-IVM imaging of the cervical spinal cord microvasculature in living mice.

From those studies using 2P-IVM to image cellular interactions in the spinal cord (14, 18), only a limited number have focused on employing 2P-IVM to study immune cell infiltration into the spinal cord during neuroinflammation (12, 19, 20). As pointed out above, all of these studies have solely focused on imaging the lower spinal cord based on the rationale that immune cell infiltration in EAE starts at the level of the lumbar spinal cord. For instance, 2P-IVM studies using a Lewis rat model of EAE and a T12/L1 spinal cord window showed that during onset of clinical EAE encephalitogenic T cells arrest in leptomeningeal vessels and crawl preferentially against the direction of blood flow before crossing the BBB (12). In the leptomeningeal space, T cells were found to be re-activated by recognition of their cognate antigen on leptomeningeal macrophages, which allowed for their subsequent crossing of the glia limitans and entering the CNS parenchyma (20, 21).

In this study, we present a novel methodology allowing for 2P-IVM of the cervical spinal cord microvasculature. This includes an advanced surgical window preparation based on a previously established cervical spinal cord window (22) as well as establishment of an anesthesia protocol enabling appropriate immobilization of the window preparation for 2P-IVM. We have optimized this methodology to specifically allow following the multistep extravasation of immune cells across the BBB including visualization of endothelial junctions in this anatomical location. Suitability of our novel methodology is exemplified by investigating the multistep extravasation of encephalitogenic T cells across cervical spinal cord microvessels during EAE.

### Summary of the Introduced Methodology

Our novel 2P-IVM imaging of the cervical spinal cord is based on the image acquisition of a surgical cervical spinal cord window prepared from the vertebrae C1 to C6. The entire sequence of procedures within the framework of this methodology, this is from pre-surgical preparation of the mice for injection anesthesia, carotid catheter and tracheal cannula insertions, inhalation anesthesia setup, head restraint, spinal cord window preparation, image acquisition, and termination of the experiment takes up to 3 h. The acquisition time can be extended up to 6 h with individual imaging sessions of 20–30 min to image the immune cell interactions with the cervical spinal cord endothelium. Equipment used in this method is optimized for C57BL/6 mice with a weight range of 18–20 g.

The current method provides a complete list of reagents, equipment and procedures required for a cervical spinal cord window preparation, and subsequent 2P-IVM imaging of the cervical spinal cord microvasculature. We also provide troubleshooting tips solving issues that may arise throughout the entire procedure. We wish to highlight at this point that performing this methodology requires basic skills in microsurgery combined with expertise in laboratory animal experimentation as well as basic knowledge in confocal and/or 2P-IVM. We recommend performing this methodology as a team of at least two researchers and/or technicians combining these skills.

### Comparison with Other Methodologies

#### Suitability of 2P-IVM versus Epifluorescence IVM in Studying Immune Cell Extravasation in the Spinal Cord

Real-time imaging with epifluorescence IVM is suitable for visualizing, e.g., rolling of immune cells, which is a fast initial step of immune cell interaction with the microvascular endothelium that typically takes place at velocities of about 10–100 µm/s. A limitation of epifluorescence IVM is the low penetration depth of about 70 µm in CNS tissues, which focuses real-time imaging rather to superficial, e.g., leptomeningeal blood vessels (2). In addition, due to potential phototoxicity and bleaching effects in epifluorescence IVM, imaging of the spinal cord needs to be interrupted between individual recording periods. An observation time of 1 min is suitable to study the initial interactions, e.g., capture and rolling, of T cells with the BBB. Firm adhesion of T cells to the BBB endothelium is typically studied by 1 min imaging intervals at several time points, i.e., 10 min, 30 min, 1 h, and 2 h after T-cell infusion. Different fields of view can typically be selected and imaged for 1 min at defined time points after infusion of T cells for later frame-by-frame off-line analysis of T cell adhesion to the BBB (8–10). Because of the intermittent imaging modus, it is difficult to determine if the respective cell followed over time is identical to the one observed in the previous imaging period.

In contrast to epifluorescence IVM, time-lapse imaging with 2P-IVM allows for long-term tracking of individual immune cells and studying the behavior of each cell. 2P-IVM is characterized by low phototoxicity, a reported tissue penetration depth of 100 and 150 µm in the CNS (15, 19) combined with good resolution (23). It thus allows for continuous imaging of slow immune cell movements such as post-arrest crawling (velocity ≈ 12 μm/min) on and diapedesis across the endothelial wall of spinal cord blood vessels (2, 12). On the other hand, owing to the single beam speed typical of most confocal settings, a single plane readout in 2P-IVM ranges from 0.5 to 1 s, therefore, tracking of fast leukocyte movements, e.g., immune cell rolling, with 2P-IVM is limited and it depends on the scanning rate achieved by the respective 2P-microscopy setup (24, 25).

In addition to the differences in imaging modalities, surgical procedures involved in spinal cord window preparations allowing for epifluorescence and 2P-IVM of the spinal cord differ significantly. To allow for 2P-IVM imaging, our previously established cervical spinal cord window preparation over vertebrae C1 to C7 suitable for epifluorescence IVM was not suitable. Rather, a significantly different cervical spinal cord window preparation needed to be developed. The more stringent requirements for tissue immobilization for 2P-IVM high-resolution imaging made it furthermore necessary to use vertebrae C1 and C6 for stabilization of the spinal cord and connection to the customized rib holders. This left C2–C5 as the exposed segment of the cervical spinal cord for 2P-IVM imaging and thus diminishes the size of the window preparation.

Moreover, owing to the extended duration of 2P-IVM imaging, laminectomy needs to be carried out without perturbing the dura mater ensuring protection of the underlying spinal cord over time. Presence of the dura mater retains the integrity of the imaged tissue and reduces the risk of injury to the spinal cord (26). However, the dura mater contains its own vascular blood supply and does not establish a BBB (27, 28). Therefore, it is advisable to remove the dura mater when imaging the spinal cord microvasculature by epifluorescence IVM to avoid interference of the dura mater microvasculature with imaging of superficial spinal cord microvessels. Tissue protection can be achieved by covering the spinal cord tissue with a transparent semipermeable membrane preventing dehydration of the exposed tissue (2).

Last but not least, anesthesia of mice allowing for 2P-IVM of the cervical spinal cord is entirely different compared with that used for epifluorescence IVM. For the latter, injection anesthesia with ketamine-hydrochloride/xylazine is sufficient to maintain deep anesthesia during the imaging (2, 22). Since ketamine does not suppress spontaneous breathing, injection anesthesia with ketamine-hydrochloride/xylazine is not suitable for the 2P-IVM imaging of the spinal cord due to breathing induced disturbance of the imaging (25). Hence, an anesthesia protocol employing isoflurane as inhalant anesthetic agent combined with ventilation was set up to improve for deep anesthesia and combined with tissue stabilization allowing for high quality of 2P-IVM imaging. Mice were shortly anesthetized by intramuscular injection of fentanyl/midazolam/medetomidine in order to place the carotid catheter, perform the tracheotomy, and connect the animal to the ventilation system. Mice were then exposed to the isoflurane inhalation before the effects of the short time anesthesia with fentanyl/midazolam/medetomidine wore off.

#### 2P-IVM Imaging Employing the Cervical Spinal Cord Window Preparation Compared to Other Spinal Cord Window Preparations

Two-photon intravital microscopy imaging of the spinal cord has been used to study the infiltration of T cells into the CNS during EAE and to this end has been limited to the lumbar and thoracic spinal cord of mice and rats (12, 19, 29).

In MS, inflammatory lesions are found in different regions of the brain and spinal cord, including the cervical spinal cord, based on the severity and the stage of disease (30, 31). Importantly, magnetic resonance imaging (MRI) of the spinal cord in MS patients demonstrated a higher frequency of cervical spinal cord lesions than thoracic or lumbar spinal cord lesions (32, 33). Recent advanced MRI imaging has pointed to a predominance of lesions in the cervical spinal cord of MS patients probably due to the fact that the cervical spinal cord has a greater cross-sectional area and contains more myelin per spinal segment than lower segments of the spinal cord (34). This study highlighted the importance of including imaging of different segments of the spinal cord in MS patients to improve understanding of disease progression and to facilitate treatment choices (34). Neuroanatomical differences of the cervical versus thoracic or lumbar spinal cord may extend to differences in angioarchitecture and thus hemodynamic parameters, which will finally impact on immune cell trafficking to the different segments of the spinal cord. Taken together, these observations underscore the necessity to expand the portfolio of spinal cord imaging techniques in animal models of EAE to the cervical spinal cord allowing to obtain new insights on immune cell entry into this segment of the spinal cord to improve our understanding of the disease pathogenesis of MS. Heterogeneity in localization of inflammatory lesions can be modeled in EAE depending on the methodology used for disease induction (35). For instance, disease processes that are induced by either γ-interferon producing Th1 or IL-17 producing Th17 cell subsets or *via* active or passive forms of EAE differ in the involvement of different parts of the CNS including the cervical spinal cord (35).

The novel cervical spinal cord window preparation introduced here is suited for 2P-IVM imaging of the cervical spinal cord microvasculature in mice during health and EAE. The window allows visualization of the area between vertebrae C2 and C5. This is different from all presently established spinal cord window preparations that allow 2P-IVM imaging of the thoracic spinal cord at the vertebral level T11 (26) or T3–T7 (29) or of the lumbar spinal cord at the vertebral level L1–L5 (27). Lumbar spinal cord windows to study T-cell interaction with leptomeningeal blood vessels have also been established in the rat at the level of T12/L1 (12, 36).

## Materials

### Mice

Female C57BL/6J mice used in this protocol were obtained from Janvier (Genest Saint Isle, France). 2D2 TCR MOG transgenic C57BL/6J mice (2D2 mice) expressing a T-cell receptor recognizing MOG_aa35–55_ in the context of MHC class II (I-A^b^) were obtained from Dr. V.K. Kuchroo (Boston, USA) (37). 2D2 mice were crossed with the transgenic mouse line with an enhanced green fluorescent protein open reading frame under the control of the human ubiquitin C promoter [C57BL/6-Tg(UBC-GFP)30Scha] (38) to create “2D2-GFP mice.” VE-cadherin-GFP knock-in mice express a C-terminal GFP fusion protein of VE-cadherin in the endogenous VE-cadherin locus, allowing for visualizing and imaging of the endothelial adherens junctions (39, 40). All mouse lines were backcrossed to a C57BL/6J background for at least eight generations and VE-cadherin-GFP mice were bred to homozygosity. Experiments were performed with 8–10 weeks old mice. Mice were housed in individually ventilated cages under specific pathogen-free conditions.

#### Anesthesia and Analgesia

- Buprenorphine: Temgesic^®^, 0.3 mg/ml (Essex-Chemie, Switzerland)
- Fentanyl: Fentanyl-Janssen^®^, 0.05 mg/ml (Janssen, Switzerland)
- Midazolam: Dormicum^®^, 5 mg/ml (Roche, Switzerland)
- Medetomidine: Domitor^®^, 1 mg/ml (Orion Pharma, Switzerland)
- Isoflurane: Attane™ (Baxter, USA)
- Carrier gas: 50% O_2_ + 50% N_2_ (Carba Gas, Switzerland).

#### Surgical Materials and Tools

- Sodium chloride for injection: NaCl 0.9% (B. Braun, Switzerland)
- Gel for covering the spinal cord: Viscotears^®^ liquid gel, Carbomer (polyacrylic acid), 2 mg/g (Alcon^®^, Switzerland)
- Eye ointment: Bepanthen^®^ (Bayer, Germany)
- Carotid catheter: Polyurethane 1 French (0.2 mm × 0.4 mm) (UNO, Netherlands)
- Suture: Silk 6-0 (Fine Science Tools, Germany), Silk 4-0 (Assut Medical Sàrl, Switzerland), Polypropylene 7-0 (Assut Medical Sàrl, Switzerland)
- Tracheal Cannula: 1.2 mm diameter (Hugo Sachs Elektronik, Harvard Apparatus, Germany).

#### Fluorescent Tracers

For contrast enhancement of blood vessels:

- Fluorescein isothiocyanate (FITC)-conjugated Dextran (MW = 150,000; Sigma-Aldrich, Switzerland)
- Texas Red dextran (MW = 70,000; Sigma-Aldrich, Switzerland)
- Alexa Fluor 594 conjugated rat-anti-mouse endoglin antibody [CD105; clone MJ7/18 (41)]. MJ7/18 was purified endotoxin-free from hybridoma culture supernatants exactly as described (42) and conjugated with Alexa Fluor 594 labeling kit (Invitrogen, Molecular probes, USA, Catalog number: A10239) according to the manufacturers instructions.

For fluorescently labeling T cells:

- Cell Tracker™ green (CMFDA; Molecular Probes, Oregon, USA)
- Cell Tracker™ blue (CMAC; Molecular Probes, Oregon, USA).

#### Equipment

- Shaver (Wahl^®^, USA)
- Electrical heating mat (Thermolux, Germany)
- TC-1000 temperature controller (CWE Inc., USA)
- Animal Bio Amp FE 136 (ADInstruments, New Zealand)
- Anesthesia Vaporizers (Hugo Sachs Elektronik, Harvard Apparatus, Germany)
- Fluovac Anesthetic Scavenging System (Hugo Sachs Elektronik, Harvard Apparatus, Germany)
- Fluosorber Canister (Hugo Sachs Elektronik, Harvard Apparatus, Germany)
- Mouse ventilator MiniVent Type 845 (Hugo Sachs Elektronik, Harvard Apparatus, Germany)
- Power lab 4/35 (ADInstruments, New Zealand)
- Stereotaxic frame; custom made by the workshop of the Theodor Kocher Institute and the Department of Chemistry and Biochemistry, University of Bern, Switzerland; www.dcb.unibe.ch/services/technical_services_and_safety/technical_workshop/index_eng.html.

#### Microscopes

- Leica M651 stereomicroscope (Heerbrugg, Switzerland).
- TrimScope two-photon laser scanning microscopy (2PM) system (LaVision Biotec, Germany) equipped with an Olympus BX50WI fluorescence microscope and a water immersion objective (20×, NA 0.95; Olympus). The 2PM system is controlled by ImSpector software (LaVision Biotec). For two-photon excitation, a Ti:sapphire laser (Mai Tai HP; Spectra-Physics) is tuned to 780–840 nm.
- Custom-made Mikron IVM500 microscope (Mikron Instruments, San Marcos, CA, USA) coupled with a 50 W mercury lamp (HBO 50 microscope illuminator, Zeiss, Switzerland) attached to the combined blue (exciter 455DF70, dichroic 515DRLP, and emitter 515ALP) and green (exciter 525DF45, dichroic 560DRLP, and emitter 565ALP) filter blocks (2).

## Methods

### Analysis of the Angioarchitecture Visible Within the Cervical Versus Lumbar Spinal Cord Window by Using Epifluorescence IVM

Cervical spinal cord windows were prepared over the vertebrae C1–C7 exactly as described before (22). Similarly, lumbar spinal cord windows were prepared at the vertebral level T12/L1 exactly as described before (12). The blood vessels of the cervical and lumbar spinal cord were visualized by contrast enhancement with 2% FITC-conjugated dextran and the use of the blue-light epi-illumination (2). FITC-dextran (MW = 150,000, Sigma-Aldrich, Switzerland) was diluted in 0.9% isotonic NaCl, pre-warmed to 37°C, and systemically applied as plasma marker by intra-carotid injection as described (8). This method allows within 1 min after injection for visualization of the entire angioarchitecture as visible in the respective spinal cord windows. Off-line analysis of the angioarchitecture, e.g., analysis of the vessels diameter, was performed exactly as described before (8, 43). Briefly, by using the CapImage software,^1^ the diameter of the spinal cord post-capillary venules was measured at distances between 90 and 100 μm from the dorsal vein (Figures 4C–E).

### Methods in Neuroimmunology

The focus of this study is on introducing the novel cervical spinal cord window preparation suitable for 2P-IVM. The two following paragraphs on induction of EAE as an example for neuroinflammation and the establishment of encephalitogenic T cells from 2D2 mice as an example for studying immune cell extravasation across the BBB in this anatomical location are kept very brief. The details of these methods are not essential for understanding the novel methodology introduced here. We rather refer to the detailed experimental protocols previously published on these methods.

### Summary of Induction of EAE

Active EAE was induced in 8–12 weeks old female C57BL/6J wild type (WT) or VE-cadherin-GFP knock-in C57BL/6 mice exactly as described before (44, 45). A detailed protocol on induction of EAE has been described before (46). VE-cadherin-GFP knock-in mice express a C-terminal GFP fusion protein of VE-cadherin in the endogenous VE-cadherin locus, allowing for visualizing and imaging of the endothelial adherens junctions (39, 40). Weights and clinical severity of EAE mice were assessed twice daily and scored as follows: 0, asymptomatic; 0.5, limb tail; 1, hind leg weakness; 2, hind leg paraplegia; 3, hind leg paraplegia, and incontinence as described in depth before (44, 47).

### Summary of CD4^+^ T cell (2D2) Isolation and Culture

In this methodology paper, T cells were isolated from the T-cell receptor transgenic 2D2 mice (37) and used as an example to study extravasation of autoaggressive T cells across cervical spinal cord microvessels by 2P-IVM imaging in the context of EAE. As any other immune cell subset can be used in this methodology, we solely refer to the vast literature describing the methodology for isolation, antigen-specific activation, and culture of 2D2 T cells here (37, 48). Methodology on testing 2D2 T cell activation, proliferation, and purity has also been described (42, 44, 48).

### Fluorescent Labeling of 2D2 T Cells

After 4 days in culture, label CD4^+^ T cells harvested from 2D2 mice with either 2.5 µM Cell Tracker™ green (CMFDA; Molecular Probes, Oregon, USA) or 20 µM Cell Tracker™ blue (CMAC; Molecular Probes, Oregon, USA) in complete medium [RPMI-1640 supplemented with 10% FBS (Thermo Fisher Scientific), 10 U/ml penicillin–streptomycin, 2 mM l-glutamine, 1% (v/v) non-essential amino acids, 1 mM sodium pyruvate, and 0.05 mM β-mercaptoethanol (Grogg Chemie AG)]. Incubate CD4^+^ T cells with either CMFDA for 45 min or with CMAC for 15 min at 37°C in the dark according to the manufacturer instructions. Subsequently, wash T cells by adding fresh complete wash buffer (HBSS supplemented with 5% FCS and 25 mM HEPES) and centrifuge for 10 min at 250 g. Directly use Cell Tracker™-labeled T cells for 2P-IVM or keep in complete medium at 37°C and 5% CO_2_ for up to 6 h before use.

Although using chemical dyes for labeling is a good technique for imaging of T cells, there are some disadvantages. For instance, using a too high concentration of Cell Tracker™ dyes disturbs the cellular function and may kill the cells (see instructions of the manufacturer). Additionally, these chemical dyes are diluted with cell divisions (2, 8). These disadvantages can be avoided by using transgenic animals that express fluorescence proteins such as GFP, which is stable and bright enough for *in vivo* imaging (23). Here, we introduce employment of CD4^+^ T cells isolated from 2D2-GFP mice. This allows visualizing the 2D2 T cells by 2P-IVM based on the expression of the GFP reporter.

### Analysis of 2P-IVM Imaging of T Cell Interactions with the Inflamed Cervical Spinal Cord Microvasculature

Two-photon intravital microscopy was used to study the arrest and post-arrest behavior of 2D2 T cells within the inflamed spinal cord microvasculature during EAE. *Arrest* of a T cell was defined as a fluorescent T cell localized at the vessel wall without moving or detaching within an observation period of at least 20 s (22). Slow intraluminal movement of polarized T cells with speeds ranging from 8 to 15 µm/min (average speed of 12 µm/min) was defined as *crawling*. T-cell crawling with and against the direction of blood flow was observed in cervical spinal cord microvessels as previously observed for T-cell crawling in lumbar spinal cord microvessels in the Lewis rat during EAE (12). *Diapedesis* of T cells across the endothelial border of the spinal cord microvessels was analyzed by using the three-dimensional (3D) Opacity renderer option of the Volocity software. Basically, the beginning of T-cell diapedesis was defined as the state when a fluorescent protrusion of the T cell appeared outside of the fluorescently labeled vascular lumen, suggesting T cell penetration across the vascular wall. T-cell diapedesis could be observed between 10 and 30 min after their arrest to the vascular wall. Completion of T-cell diapedesis was defined when the entire fluorescently labeled T-cell body could be detected outside the vascular lumen.

Our *in vitro* studies have shown that T-cell diapedesis across the BBB can occur through the endothelial junctions, referred to as paracellular diapedesis, or through the endothelial cell body, which is referred to as transcellular diapedesis (39, 49). To this end, we explored visibility of endothelial junctions in spinal cord microvessels in VE-cadherin-GFP knock-in mice by 2P-IVM and found that 2P-IVM allows defining endothelial junctions in this vascular bed by the localization of the GFP reporter. Thus, VE-cadherin-GFP knock-in mice are suitable to explore the cellular pathway of T-cell diapedesis across the BBB *in vivo*.

### Procedures of Mouse Preparation for Anesthesia, Cervical Spinal Cord Window Surgery, and Image Acquisition

#### Pre-surgical Preparation of Mice for Injection Anesthesia—1 h

**Figure 1:**
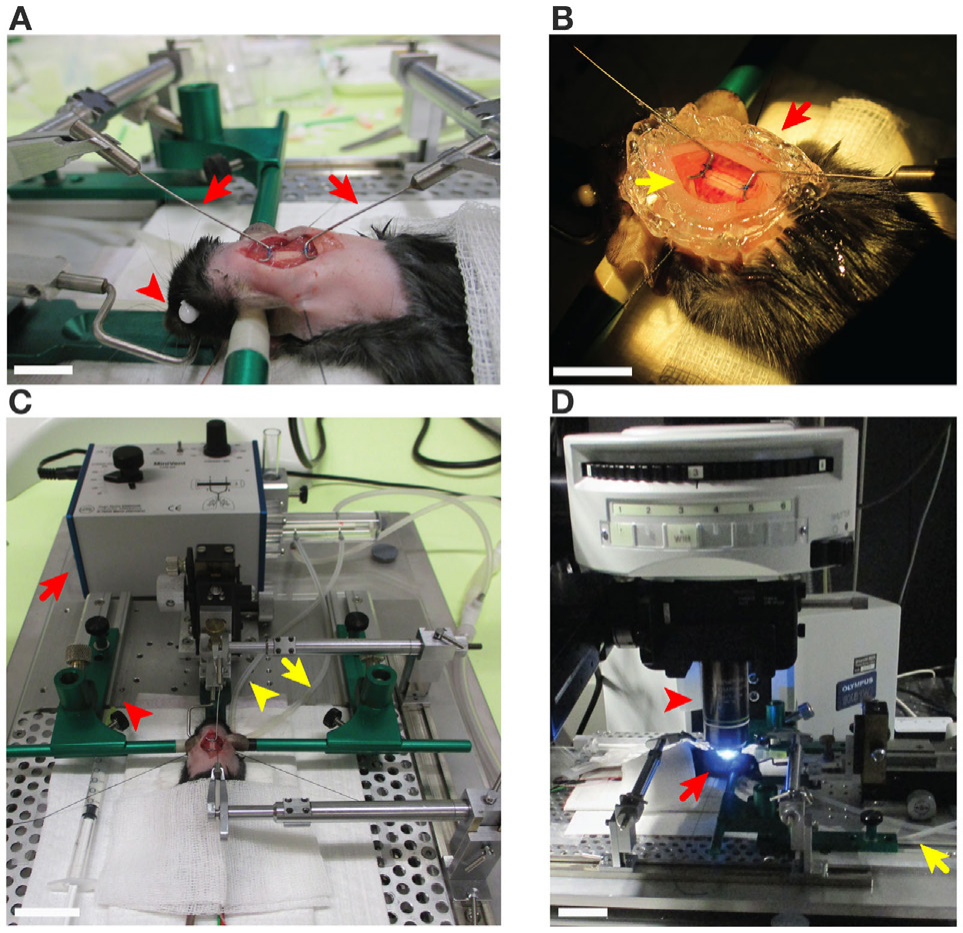
**Preparation steps of the cervical spinal cord window in C57BL/6 mice prior to two-photon intravital microscopy imaging**. **(A)** An anesthetized mouse on the stereotaxic frame from the left side described in steps 3, 13, and 15 of the procedures. The ear bars (green) were gently placed into the ear canals in order to tightly immobilize the head of mouse. The eyes of the mouse were covered with an ophthalmic ointment (red arrowhead) to prevent irritations of the cornea. The fur around the neck (2–2.5 cm caudal) was shaved for the spinal cord surgery. The cervical spinal cord window was prepared and is stabilized at C1 and C6 with two customized rib holders (red arrows) that are fixed on the stereotaxic frame. Scale bar: 10 mm. **(B)** The surgically prepared cervical spinal cord has been sealed with 1% agarose or grease (red arrow) to maintain a pocket for the Viscotears^®^ liquid gel (yellow arrow) for the water immersion objective of the microscope (step 15 of the procedures). Scale bar: 10 mm. **(C)** The mouse on the stereotaxic frame was connected to the MiniVent ventilator (red arrow) *via* the tracheal catheter (yellow arrowhead) allowing for isoflurane inhalation anesthesia by providing a mixture of isoflurane and O_2_/N_2_ (steps 5, 7, and 13 of the procedures). Waste gas was deflected (yellow arrow) and transfered to the scavenger system (step 8 of the procedures). Pre-warmed T cell suspension and blood vessel marker were infused *via* a syringe to the carotid catheter (red arrowhead) as described in step 22 of the procedures. Scale bar: 30 mm. **(D)** The preparation was transferred to the two-photon microscope and the cervical spinal cord (C2–C5) was exposed as described in step 17 of the procedures (red arrows). The MiniVent ventilator connected *via* tracheal catheter (yellow arrow) to the anesthetized mouse was moved with the mouse to the two-photon microscope stage. The interaction of T cells with cervical spinal cord microvessels was scanned using 20× water immersion objective (red arrowhead). Scale bar: 10 mm.

1. Check the EAE animals and score them. Mice showing clinical scores from 0.5 (limp tail) to 2 (hind leg paraplegia) and with a body weight of at least 15 g are preferably used for 2P-IVM experiments as they tolerate the anesthesia very well compared to mice with higher disease scores.
2. Weigh the animal and administer a single dose of buprenorphine (Temgesic; 0.375 mg/kg body weight) subcutaneously. Buprenorphine is an analgesic drug applied to provide adequate pain relief while the mouse receives inhalation anesthesia (isoflurane). It should be injected at least 30 min prior to the tracheotomy and isoflurane administration. In order to perform the tracheotomy (described in points 6–8), the mouse is anesthetized *via* intramuscular injection of fentanyl (0.05 mg/kg)/midazolam (5 mg/kg)/medetomidine (0.5 mg/kg). These drugs show a rapid onset and short duration of action. Fentanyl^2^ is a synthetic opioid analgesic, the sedative midazolam^3^ is a short-acting benzodiazepine derivative, and medetomidine is a synthetic adrenoreceptor agonist with sedative and analgesic properties.^4^
3. Shave the fur on the neck and upper back. Carefully monitor the anesthetic state of the mouse by checking awareness signs such as paw withdrawal reflex, blinking reflex, and whisker twitching. Proceed with fur shaving on the neck using an electrical shaver when the mouse shows a sustained loss of reflexes (Figure 1A).
4. Apply Bepanthen (an ophthalmic ointment) to the eyes to prevent irritations of the cornea.
5. Position the mouse on the stereotaxic frame (modified and custom-designed from David Kopf Instruments, USA and Narishige accessories for Stereotaxic, Japan) (Figures 1A,C). Move the stereotaxic frame under the stereomicroscope for the surgery of cervical spinal cord window.

#### Carotid Catheter and Tracheal Cannula Insertions—30 min

6. A carotid catheter is placed allowing for systemic injection of exogenously labeled cells or fluorochrome-labeled plasma markers or antibodies *via* the arterial blood supply and thus immediate imaging as described before (2, 8). Position the mouse under a dissecting stereomicroscope in dorsal recumbency and make a 1.5 cm long ventral cervical skin incision between the upper thorax aperture and the lower jaw. Separate the tissue by using angulated blunt dissection forceps to visualize the right carotid artery. Wrap sutures (silk, size 6-0) loosely around the vessel and insert a polyurethane catheter (size 1 French) into the carotid artery pointing toward the aortic arch. Secure the cannula with two prepared sutures and verify its patency by injection of 0.9% NaCl.
7. Continue with performing a tracheotomy before closing the skin incision. Perform tracheotomy to insert the tracheal cannula and connect to the ventilator. To achieve that, separate the lobes of the thyroid gland bluntly at their isthmus. To expose the larynx and the trachea, spread the sternohyoid muscles bluntly and pull them aside. Conduct the tracheotomy by creating an opening with a microscissor between the second and the third tracheal rings caudal from the larynx. Insert the cannula into the trachea and secure it in place by one or two ligatures around the trachea. Close the incision with sutures. Lastly, connect the tracheal cannula *via* a catheter to a mouse ventilator (MiniVent) (Figure 1C).

#### Inhalation Anesthesia Setup—10 min

**Figure 2:**
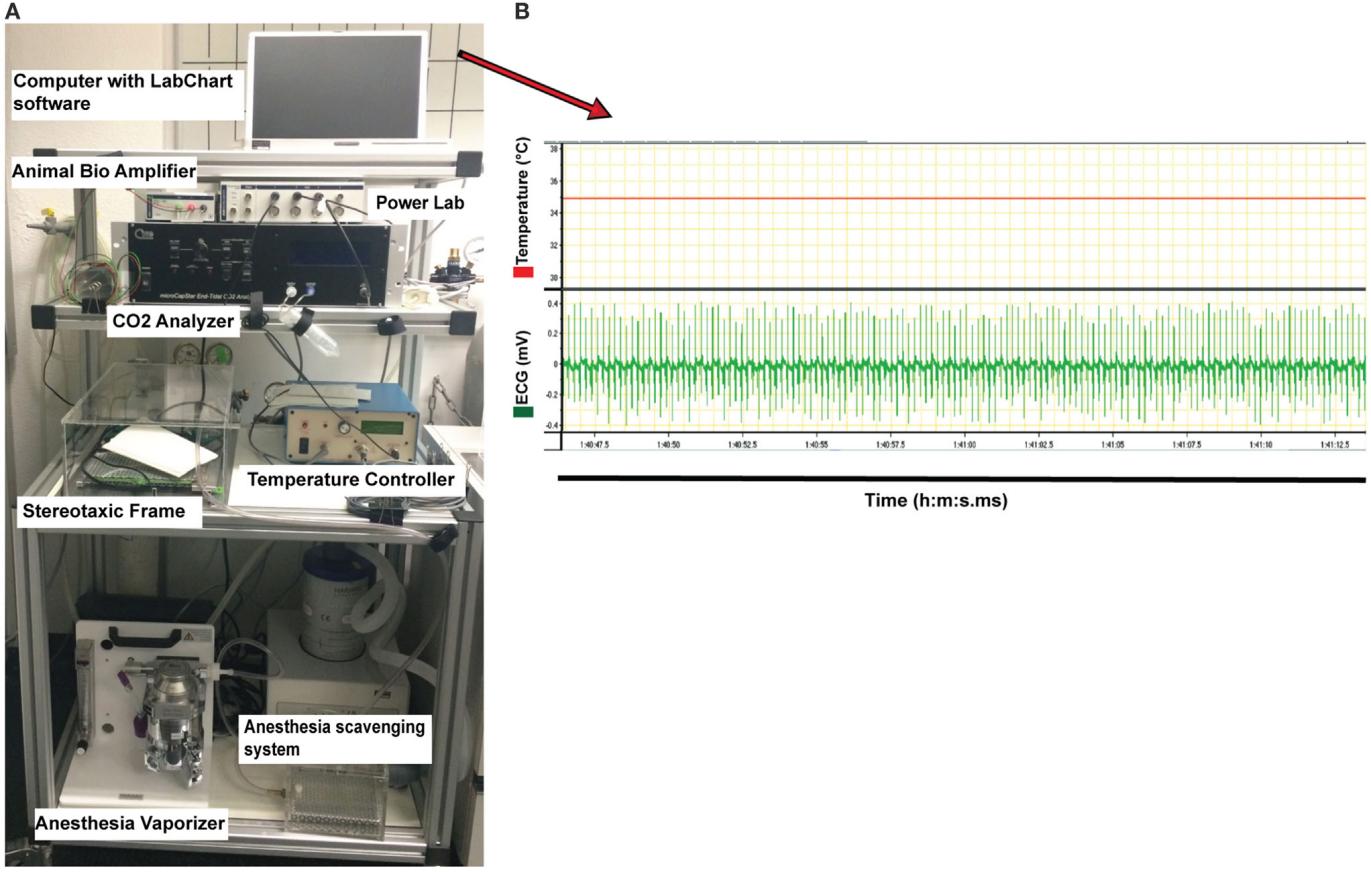
**Instrument setup used for monitoring physiological parameters of the anesthetized mouse during two-photon intravital microscopy imaging of the cervical spinal cord**. **(A)** The instruments required for anesthesia and for controlling the physiological parameters of the mouse (all placed on a cart allowing for moving) are shown. This includes the following: vaporizer for the anesthesia gas (isoflurane), anesthesia scavenging system for the removal of waste isoflurane, temperature controller machine for monitoring the temperature, CO_2_ analyzer measuring exhaled CO_2_ levels, Animal Bio Amp for electrocardiogram (ECG) measurement, and PowerLab data acquisition hardware for transferring the signals to the computer with LabChart software. **(B)** Example of the original diagrams provided by LabChart software during measuring the temperature and ECG and stored on the laptop computer (showed in **(A)**, on top of instrument setup picture). Stable body temperature of 35°C (red line, top diagram) and the regular ECG (mV) of the mouse (green line, below diagram) recorded *via* the Animal Bio Amplifier during 25 s are shown. The temperature of the mouse was maintained at 35 ± 1°C during the entire surgery by the temperature controller.

**Figure 3:**
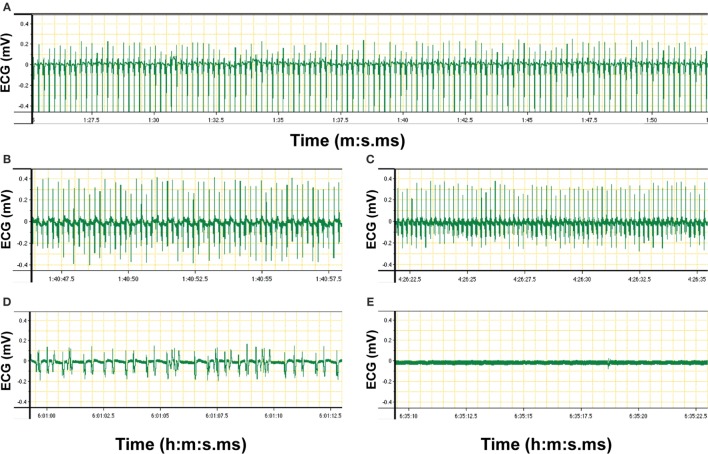
**Electrocardiogram (ECG) signal of an anesthetized C57BL/6 mouse recorded via LabChart software (original diagrams are displayed)**. Electrical activity of the heart of the mouse was monitored *via* Animal Bio Amp devices. Signals were transferred *via* PowerLab hardware to the LabChart software on the computer. The ECG graph showing voltage over time was produced by the LabChart software and stored on the computer. **(A)** Recording of ECG signals was started at 1 min:27 s 5 ms (1:27.5) after the connection of the electrodes to the mouse. The ECG graph shows a recording from 1:27.5 to 1:50 (minute:second.millisecond), i.e., a measurement over 23.5 s. Regular ECG was an indicator of the stable condition of the anesthetized mouse as prerequisite for two-photon intravital microscopy imaging. **(B,C)** Changes in ECG rhythms were monitored over the time of imaging. **(D)** Irregular ECG indicates unstable heartbeat of the anesthetized mouse. **(E)** Flatline is recorded if there is no electrical activity of the heart of the mouse and thus indicates the death of the animal.

8. Turn on the anesthesia vaporizer, anesthesia gas scavenger system, and the ventilator machine. Before the effects of the short time anesthesia with fentanyl/midazolam/medetomidine (described in step 2 of the procedures) wear off, the mouse must be exposed to isoflurane inhalation. Protocols provided by the manufacturer of the vaporizer used in this study have suggested the use of 1% isoflurane for healthy mice with a body weight of 25–30 g (50) or of 1.5–3% isoflurane for mice with 28–32 g weight (51). Taking into account the low body weight of mice suffering from EAE (15–18 g) and their disease status, we have tested isoflurane levels between 0.5% and 2% and found 0.5–1% isoflurane to be optimal for animals in this condition. Therefore, we suggest to ventilate the mouse with 0.5–1% isoflurane in a mixture of 50% O_2_ and 50% N_2_ applying a tidal volume of 175 µl and a respiration rate of 120 strokes/min to maintain general anesthesia. The gas scavenger system reduces the risk of exposure to isoflurane from the surgical area (Figure 1C). Carefully monitor the level of anesthesia during the entire process of surgery and imaging. Ensure a deep level of anesthesia by performing toe pinch and checking paw withdrawal reflex every 60 min. Increase the supply level of isoflurane to 1.5–2%, in case of any observed reflexes or whisker twitching, which are the indicators of insufficient anesthesia.
9. Use the electronically regulated animal-heating pad that is connected to the temperature controller machine. Use the rectal probe attached to a digital thermometer (temperature controller) to monitor the body temperature, which should be maintained at 35 ± 1°C. Securely tape the rectal probe to the heating pad for easier handling.
10. Carefully monitor the electrical activity of the heart by electrocardiogram (ECG) during the spinal cord surgical process and imaging acquisition. Place the ECG electrodes under the skin of one leg and chest of the mice for screening during the experiment.
11. Start recording of temperature and ECG signals using the LabChart software (ADinstruments, New Zealand) (Figures 2 and 3A–E).

#### Head Restraint—20 min

12. Change the position of the mouse to the prone position in a stereotaxic frame. Turning should be done very carefully to prevent kinking of the carotid and tracheal catheter and displacement of the ECG electrodes and rectal probe.
13. Raise the head of the mouse to place it in a stereotactic head holder. Fix the teeth of the mouse on the tooth holder and place the tongue outside of the mouth to avoid suffocation. Loosen the screws of one ear bar and slide the ear bar gently into the ear canal. Do not push the ear bars into the canal with too strong force. Tighten the screws and follow the same procedure for the other ear bar. Place the head horizontally in line with the entire body and tightly immobilize between the ear bars (Figures 1A,C).

#### Spinal Cord Window Preparation—45 min

14. Injection of NaCl solution: to compensate for fluid loss and to maintain well-being of the mouse during surgery and the subsequent imaging session, inject a subcutaneous bolus of 1.0 ml of 0.9% NaCl in the back.
15. Make a midline skin incision on the neck 1.5–2 cm long, from the occiput to the cervical–thoracic transition (Figure 1A). Cut the paravertebral musculature longitudinally and retract to both sides by sutures. After identifying the lamina of the sixth cervical vertebral arch, carry out a laminectomy from C1 to C6 under the stereomicroscope, avoiding damage to the underlying tissue. Make sure to keep the dura mater intact to protect the underlying spinal cord tissue. Remove the lamina from C2 to C5 using curved surgical scissors and employ C1 and C6 for stabilization and suspending of the spinal cord window. Use polypropylene sutures 7-0 to tie knots on C1 and C6 and connect them to customized rib holders attached to the stereotaxic frame (Figure 1A). Cover the spinal cord window with Viscotears^®^ liquid gel suitable for water immersion objectives (Figures 1B,D).
16. Spinal cord preparations showing surgical trauma (e.g., bleeding) or any signs of acute inflammation, such as hyperemia, stagnant blood flow, or distorted vessels (22), were excluded from further imaging experiments.

#### Image Acquisition—1–6 h

17. Transfer the immobilized mouse in the stereotaxic frame and the MiniVent ventilator to the two-photon microscope stage. If you need to transport the preparation from the stereotaxic microscope to the two-photon microscope over a longer distance, pay specific attention to avoid displacing of the heating probe, ECG electrodes, carotid catheter, and tracheal catheter, which are connected to the anesthetized mouse (Figure 1D).
18. Set the appropriate laser wavelength (780–840 nm).
19. Adjust the appropriate laser power (20–50 mW).
20. To image the blood vessels there are two different options.
  20.1. For imaging intact blood vessels, infuse a fluorescent plasma marker or fluorescent anti-endothelial antibody *via* the carotid catheter using a 1 ml syringe. High molecular-weight fluorescently labeled dextrans (MW = 70,000–150,000) are suitable for visualizing blood vessels. Here, we used as an example Texas Red dextran (MW = 70,000, 10 µg/mouse). Alternatively, Qtracker^®^ non-targeted quantum dots can be used as plasma markers. Quantum dots are semiconducting nanocrystals with specific fluorescent characteristics depending on their size. Qtracker^®^ quantum dots have been optimized for *in vivo* imaging by a polyethylene glycol (PEG) surface coating.^5^ The PEG coating minimizes non-specific interactions of the Qtracker^®^ quantum dots in the blood stream and due to the lack of reactive functional groups extends the circulation time of the Qtracker^®^ quantum dots to several hours allowing for high quality vascular imaging over extended time.
  20.2. For imaging CNS microvessels during neuroinflammatory disorders like EAE, injection of fluorescent dextran or quantum dots may not be feasible in case an impaired BBB will allow their leaking out and blurring the image and thus prohibiting subsequent 2P-IVM imaging of the vasculature. In this case, fluorophore-conjugated endotoxin-free anti-endothelial antibodies can be infused to label the vascular endothelial cells and thus allow outline of the vascular wall *in vivo*. Here, we introduce injection of Alexa Fluor 594 labeled rat-anti-mouse endoglin (CD105) antibody (2 µg/mouse). This antibody does not interfere with immune cell interaction of the CNS microvessels as shown before (2, 8).
21. Evaluate the quality of the preparation by scanning the entire cervical spinal cord window. Exclude the blood vessels with surgical trauma that may have inadvertently occurred during the surgical procedure. Leakage of the fluorescent plasma marker out of the blood vessels indicates BBB breakdown, which is a pathophysiological hallmark of EAE. In this case, use anti-endothelial antibodies to visualize the vascular lumen as described in step 20.2 of the procedures.
22. Re-suspend the desired number of T cells in a small volume (maximum 300 µl) of 0.9% isotonic NaCl. Fill the pre-warmed T cell suspension (37°C) into a 1-ml syringe and systemically inject the T cells *via* the carotid catheter.
23. Scan the entire cervical spinal cord window to select appropriate regions of interest (ROI). For orientation, use the central posterior vein visible in the midline of the dorsal spinal cord, which drains the blood caudally. The function of this vessel is analogous to that of the superior sagittal sinus in the cerebral microcirculation. There are capillaries and post-capillary venules visible on both sides of the dorsal vein, which drain into this posterior vein.
24. Choose a ROI in order to image the interaction of T cells with the BBB endothelium in the leptomeninges or deeper in the spinal cord white matter. Use the second harmonic generation (SHG), which is visualizing collagen fibers to adjust the orientation and set a zero level (basal level). SHG is a non-linear coherent scattering process that conserves energy (23). In the spinal cord window, presence of collagen is limited to the leptomeninges and thus visibility of SHG defines the imaging levels to the leptomeninges.
25. Set the *z*-space and the region of imaging. The *z*-steps of 2–4 µm are appropriate to study the T-cell interaction with the microvessels.
26. Define a time interval of scanning.
27. Acquire 4-dimensional (4D) images starting at 15 min after T-cell injection. Perform the image acquisition using the ImSpector software (LaVision Biotec) or equivalent software. For 4D(*x, y, z*, and time) analysis of T-cell migration, acquire 10–15 *x*–*y* sections (200–400 µm × 200–400 µm scan field) with *z*-steps of 2–4 µm (20–60 µm total scanning space) every 20 s for 20–30 min. To generate three-color images, in the present example, we collected the emitted light and second harmonic signals through 447/55-nm (for SHG, CMAC), 525/50-nm (for GFP, CMFDA), and 593/40-nm (for Texas Red, Alexa Fluor 594 conjugated rat-anti-mouse endoglin antibody) bandpass filters with non-descanned PMT detectors.
28. Repeat steps 24–27 for each ROI.
29. Transform sequences of image stacks into volume-rendered 4D image sequences with appropriate software (e.g., Volocity from Perkin Elmer or Imaris from Bitplane), which is also used for semi-automated tracking of cell motility in three dimensions. For visualization, adjust the image sequences for brightness, contrast, and background noise by using appropriate software and then export the image sequences in QuickTime format.

#### Termination of Experiment—5 min

At the end of the *in vivo* imaging, the mouse can either be euthanized or tissues can be prepared for further histological or morphological analysis.

30. Euthanization of the mouse: At the end of the experiment, sacrifice the mouse by intra-arterial application of an overdose of ketamine/xylazine *via* the carotid catheter.
31. Alternatively, to prepare the spinal cord tissue for further analysis perfuse the mouse with 15 ml 1% or 4% paraformaldehyde in phosphate buffered saline *via* the carotid catheter. Collect spinal cord tissue and process the tissue according to the methodology chosen for further analysis, e.g., for immunofluorescence microscopy.
32. For immunofluorescence microscopy, carefully embed the spinal cord tissue in Tissue-Tek (Sakura Finetek, Netherlands) and snap freeze in a 2-methylbutane bath cooled with dry ice to –80°C in a Dewar vessel exactly as described (22).

## Troubleshooting Tips

**Table:** 

Procedure steps no.	Problem	Possible explanation	Solution
2	Mouse with experimental autoimmune encephalomyelitis (EAE) dies after injection of fentanyl/midazolam/medetomidine	The mouse is too ill or lost too much weight during EAE and cannot tolerate anesthesia.	Do not use EAE animals with a clinical score more severe than paraplegia or with weight loss of more than 20%.

2	Animal is not anesthetized after 10–15 min of fentanyl/midazolam/medetomidine injection	Anesthetic mixture was not properly injected intramuscularly.Anesthetic has lost its potency, as it is either expired or has not been freshly prepared.	Repeat administration with a half of the original dosage.Open a new stock or freshly prepare the anesthetic mixture.Store anesthetics in lightproof containers.

7	Observing signs like twitching of whiskers showing that the animal begins to wake up after connection to the ventilator	Diameter of the tracheal cannula is too small for the diameter of the trachea or has not been appropriately fixed with the sutures. Consequently, isoflurane is leaking out.	Make sure that the outside diameter of the tracheal cannula fits tightly within the animal’s trachea and fix appropriately.

8	Overdose of anesthetics	Time interval between the first anesthetic (fentanyl/midazolam/medetomidine) and the second one (isoflurane) is too short.	After connection of the animal to the ventilator, first apply only mixed gas of O_2_ and N_2_. Apply isoflurane only after a proper time interval.

10	Irregular electrocardiogram	Animal did not receive a proper volume of anesthetics.The electrodes have been dislocated.	Increase the isoflurane percentage (suggested appropriate level: 0.5–1%).Check the electrodes and be sure that they are connected at the corresponding positions.

9	Body temperature is not stable and temperature controller gives an alarm	Rectal probe for temperature has not been inserted properly.	Check the rectal probe and secure it with tape. Temperature should be set on 35 ± 1°C.

12	Abdomen of the mouse is inflated after turning to the prone position	Trachea or tracheal catheter has been twisted by turning the mouse and blocked the flow of anesthesia gas to the lung.	Repeat the surgery with a new animal. To avoid inflation, turn the animal and all the tubing connections at the same time and in the same direction.

13	Ear bars do not engage the ear canal	Position of animal’s head is not straight.Head is in a higher or lower level compared to the ear bars.	Place the head in a horizontal position in line with the body and avoid bending.Add or remove gauze pads under the belly of the mouse.

15	Bleeding	Damage to the blood vessels during surgery.	Use forceps with an angle and use them gently.

15	Cervical bones are broken while stabilizing the spinal cord	Excessive pressure was applied.	Avoid putting too much pressure on the preparation while stabilizing the bones with rib holders.

16	No observation of cells or blood flow in the microcirculation of the spinal cord window after infusion of cells *via* the carotid catheter	Cells might have aggregated and blocked blood flow in a blood vessel outside of the regions of interest (ROI).	Check additional fields of view. Repeat with a second injection.If not successful start with a new animal. To avoid aggregation of cells, carefully resuspend cells in 0.9% isotonic NaCl right before infusion to the catheter.

17	Blood flow in dorsal vein is slowed down	Body temperature of the animal is dropping.	Cover the animal with some gauze pads. Check if the rectal temperature probe is properly inserted and whether the temperature controller is adjusted correctly.

21, 23	ROI is not visible under the two-photon microscope	Viscotears^®^ liquid gel is leaking out of the window preparation.	Apply agarose around the whole spinal cord window to avoid leaking of Viscotears^®^.

18, 19	Fluorescence signals are low	Laser power and the voltage of PMTs are low.Excitation wavelength is not appropriate.Bleeding from surrounding tissue covered the ROI.Agarose has covered ROI.	Increase the laser power and the respective PMT voltages.Change to another excitation wavelength.Clean the blood from ROI with a gauze pad and NaCl.Clean ROI with gauze pad and NaCl.

20	Fluorescence signal of blood vessels is low	Fluorescent dextran dye or fluorescently labeled antibody has leaked from the vessels.	Repeat injection of plasma marker. If there is no improvement repeat the whole surgical procedure with a new animal.

## Results

**Figure 4:**
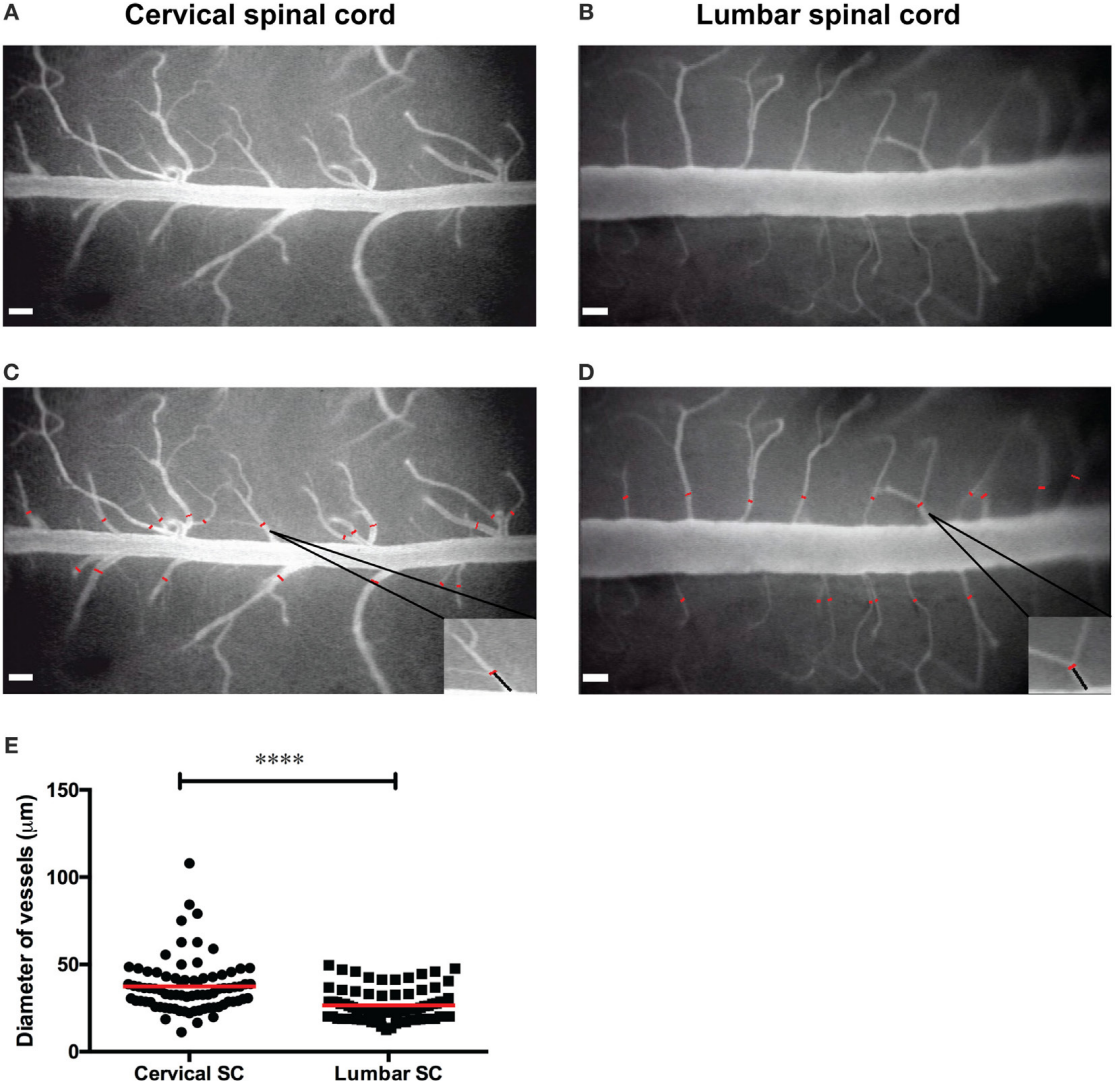
**Angioarchitecture in the lumbar versus cervical spinal cord of healthy C57BL/6 mice**. **(A,B)** Representative epifluorescence intravital microscopy images of the cervical **(A)** and the lumbar **(B)** spinal cord window preparations and analysis of vessel diameters are shown. Contrast enhancement of the spinal cord microvasculature was obtained by injection of 2% fluorescein isothiocyanate-conjugated Dextran and blue-light illumination using a 4× long-distance objective. **(C,D)** Off-line measurement of vessels diameters was performed using CapImage software (described in Section “Methods”). The diameters of the post-capillary venules (red lines) were measured at a distance of 90–100 µm from the dorsal vein as indicated for one example each and marked by the black line in the inset. **(E)** The diameters of the post-capillary venules in the cervical spinal cord window and lumbar spinal cord window were compared. Each dot represents one post-capillary venule. The diameter of 79 post-capillary venules from 3 animals per group was analyzed. Quantification was done by using the GraphPad Prism software (version 6.00, CA, USA). Statistical significant was determined by the Mann–Whitney *U*-test. Data are presented as mean value ± SEM. Asterisks indicate significant differences (****p < 0.0005). Scale bar in panels **(A–D)**: 100 µm.

We first assessed the differences of the dorsal spinal cord angioarchitecture visible by epifluorescence imaging when using established cervical and lumbar spinal cord window preparations, respectively. To this end, we used our previously established cervical spinal cord window preparation over the vertebrae C1–C7 (8) and the lumbar spinal cord window preparation at the vertebral level T12/L1 (12). The dorsal spinal cord angioarchitecture was imaged by epifluorescence IVM 1 min after their visualization by intra-arterial injection of FITC-dextran and its homogenous distribution throughout the visible vasculature. As previously observed, the cervical spinal cord angioarchitecture is characterized by a collecting vein localized in the dorsal midline of the spinal cord draining blood in a caudal direction (Figures 4A,C). The function of this vessel is analogous to that of the superior sagittal sinus in the brain circulation. In the lumbar spinal cord window (Figures 4B,D), this collecting vein was characterized by its significantly larger diameter of ±235 µm compared to its diameter of ±130 µm in the cervical spinal cord and drainage of blood in cranial rather than caudal direction. In contrast, in the cervical spinal cord the average diameter of post-capillary venules draining blood toward the dorsal vein was significantly larger (37.50 ± 1.74 μm; *n* = 79; Figure 4E) when compared with that of the post-capillary venules in the lumbar spinal cord (26.52 ± 1.025 μm; *n* = 79; Figure 4E). In the lumbar spinal cord, these post-capillary venules were oriented in a perpendicular or in a caudal to cranial fashion toward the dorsal vein. Their orientation and direction for blood flow was strictly proximal to caudal in the cervical spinal cord (Figures 4A,B). Importantly, in both window preparations, arteries were not visible as previously described (8, 28). According to the Hagen–Poiseuille law that assumes a parabolic flow profile in blood vessels, the observed differences in the diameters of the cervical versus lumbar spinal cord post-capillary venules will translate into differences of the hemodynamic parameters present in the different post-capillary vascular beds, which finally impact on the multistep T cell interaction with the BBB in the different regions of the spinal cord (2, 8, 52).

We next used our novel cervical spinal cord window preparation for 2P-IVM to image the interactions of auto-aggressive CD4^+^ T cells with inflamed spinal cord microvessels during EAE. CD4^+^ T cells were harvested either from 2D2 GFP mice or from 2D2 mice and then fluorescently labeled with Cell tracker™ dyes as described in the experimental setup above. Using GFP^+^ T cells or CellTracker labeled T cells allowed us to visualize transferred T cells and to follow their multistep interaction with the cervical spinal cord microvessels during EAE *in vivo*.

Laminectomy was applied in 8–10 weeks old anesthetized WT or VE-cadherin-GFP knock-in mice suffering from EAE with a clinical score between 0.5 (limp tail) and 2 (hind leg paraplegia). Anesthesia, temperature, and heart activity of the mice were monitored during all preparation steps. After preparation of a cervical spinal cord window, the preparation on the stereotaxic frame was moved to the two-photon microscope stage (Figure 1D). If the stereomicroscope and the two-photon microscope are not in the same room, this involves of course moving the MiniVent ventilator connected *via* the catheter to the trachea of the anesthetized mouse, as well as the tracheal catheter, the heating probe, ECG electrodes, and carotid catheter all connected to the immobilized mouse in the stereotaxic frame. In this case, it is advised to place the entire equipment on a movable cart as shown in Figure 2A. Plasma marker or fluorescent antibody was systemically injected *via* a syringe into the carotid catheter of the surgically prepared mouse to allow for contrast enhancement of the spinal cord vasculature. Any blood vessel damage occurring during surgical preparation of the cervical spinal cord window will cause massive leakage of the fluorescent dye or the antibody. Therefore, only an intact microcirculation without bleeding was considered for further 2P-IVM analysis of encephalitogenic CD4^+^ T cell interactions with the BBB. During EAE, BBB leakiness is a pathophysiological hallmark why using soluble plasma tracers might not be feasible. In this case, injection of a fluorescently labeled anti-endothelial antibody, e.g., anti-endoglin antibody as outlined above (step 20.2 of the procedures) that does not interfere with T-cell interaction with the BBB is the method of choice (8, 9).

A pre-warmed T cell suspension (37°C) consisting of 5–10 × 10^6^ 2D2 GFP or Cell tracker™-labeled 2D2 T cells in 300 µl of 0.9% NaCl solution was systemically injected *via* a 1-ml syringe into the carotid catheter of a surgically prepared mouse. The entire cervical spinal cord window was scanned and ROIs were chosen for the imaging after adjusting the zero level (basal level) of scanning based on the prominent SHG signal. SHG signal was observed due to the presence of collagen fibers in the leptomeninges. Images were acquired from an area 200–400 µm × 200–400 µm × 20–60 µm, starting typically at a depth of >50 µm below the surface identified by SHG signal. The behavior of CD4^+^ T cells, such as arrest, crawling, and diapedesis, was imaged over a period of 20–30 min. It is necessary to image the target many times in the *z*-stacks for a proper 3D analysis (17, 25). Activated T cells had a size of 10–12 µm, therefore, we mostly adjusted the 4 µm *z*-steps in order to detect T cells at least 2–3 times in multiple layers of the *z*-stacks.

**Figure 5:**
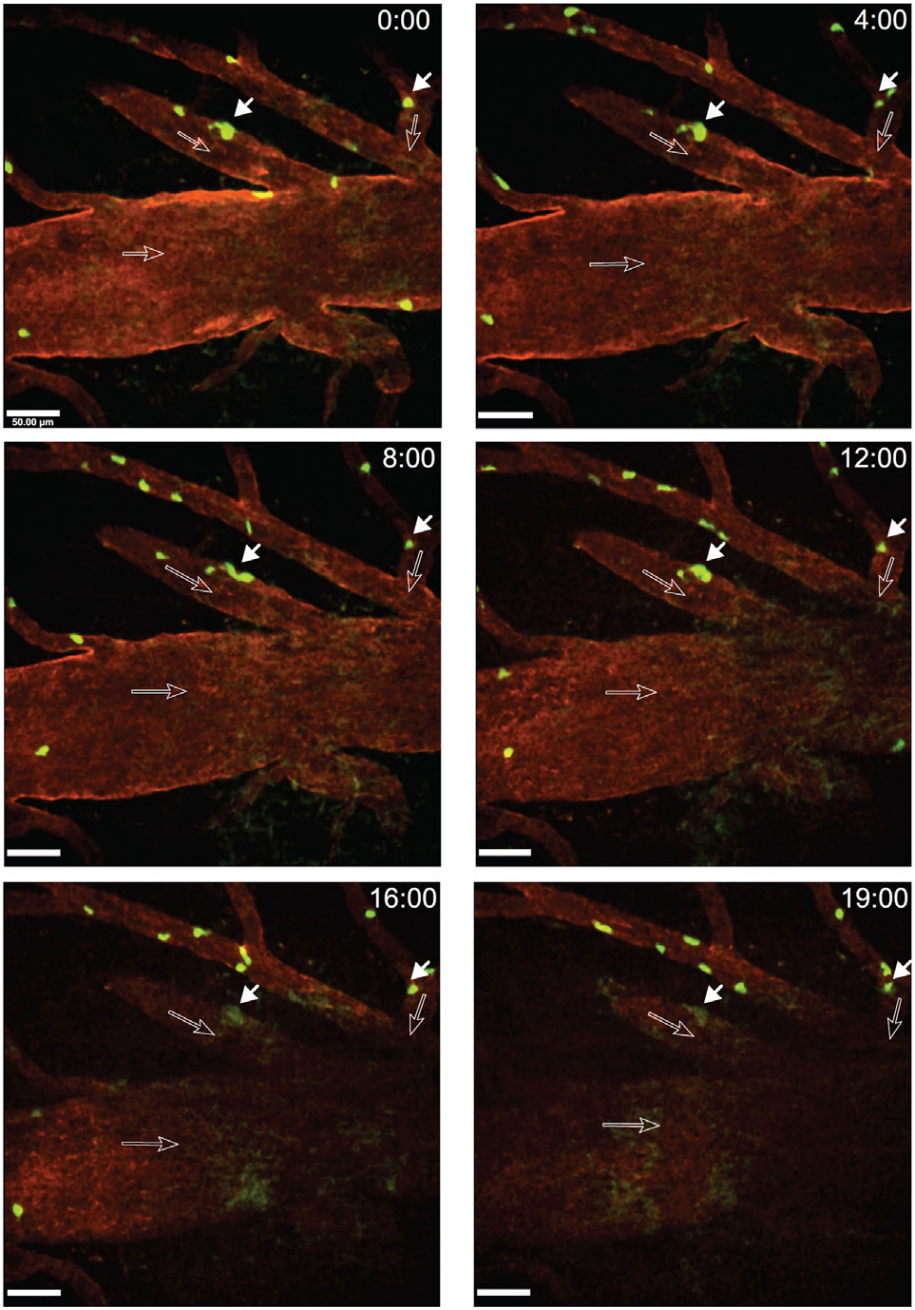
**Arrest of activated 2D2 GFP CD4^+^ T cells within inflamed cervical spinal cord post-capillary venules during experimental autoimmune encephalomyelitis (EAE)**. Active EAE was induced as described in Section “Methods.” Laminectomy was performed on day 14 post-immunization when the mouse showed onset of disease. *In vitro* activated CD4^+^ T cells from 2D2 GFP mice were injected *via* a carotid catheter before two-photon intravital microscopy (2P-IVM) imaging. Blood vessels were labeled by injection of Alexa Fluor 594 conjugated anti-endoglin antibody. GFP (green, CD4^+^ T-cells) and anti-endoglin (red, blood vessels) were excited at 800 nm using a tunable MaiTai HP laser (Spectra Physics). The dorsal vein is visible in the middle of the regions of interest (ROI). A *x*–*y*–*t* time-lapse sequence of a 400 µm × 400 μm scan field at a depth of 59–91 µm and 9 *z*-stacks with 4 µm spacing shows the arrest of CD4^+^ T cells within post-capillary venules of the cervical spinal cord. Time is shown in minutes and seconds. Filled arrows show CD4^+^ T cells (green) arrested on the luminal surfaces of the spinal cord microvessels and open arrows show the direction of blood flow. The respective positions of the arrested T cells are shown at 0, 4, 8, 12, 16, and 19 min of recording (Video S1 in Supplementary Material). Arrested T cells did not move or detach from the vessel wall within a time frame of 20 s. Due to minute movement of the mouse, a part of the ROI fades out of focus after 16 min of recording. Scale bar: 50 µm.

A fraction of CD4^+^ T cells was observed to arrest on the luminal surface of the spinal cord microvessels without further moving and eventually to detach from the vessel wall within an observation period of at least 20 s (Figure 5; Video S1 in Supplementary Material). When observing a total of 13 CD4^+^ T cells interacting with the post-capillary venules in a movie of 20 min we found three of these T cells arrested on the BBB endothelium and the remaining 10 T cells crawled on the luminal side of the vasculature or detached from the vessel wall and were washed away with the blood flow. In another mouse, we observed 25 CD4^+^ T cells within one ROI in a movie of 9 min. In this case, 15 T cells arrested on the vessel wall and did not move within an observation of at least 20 s (Video S2 in Supplementary Material).

**Figure 6:**
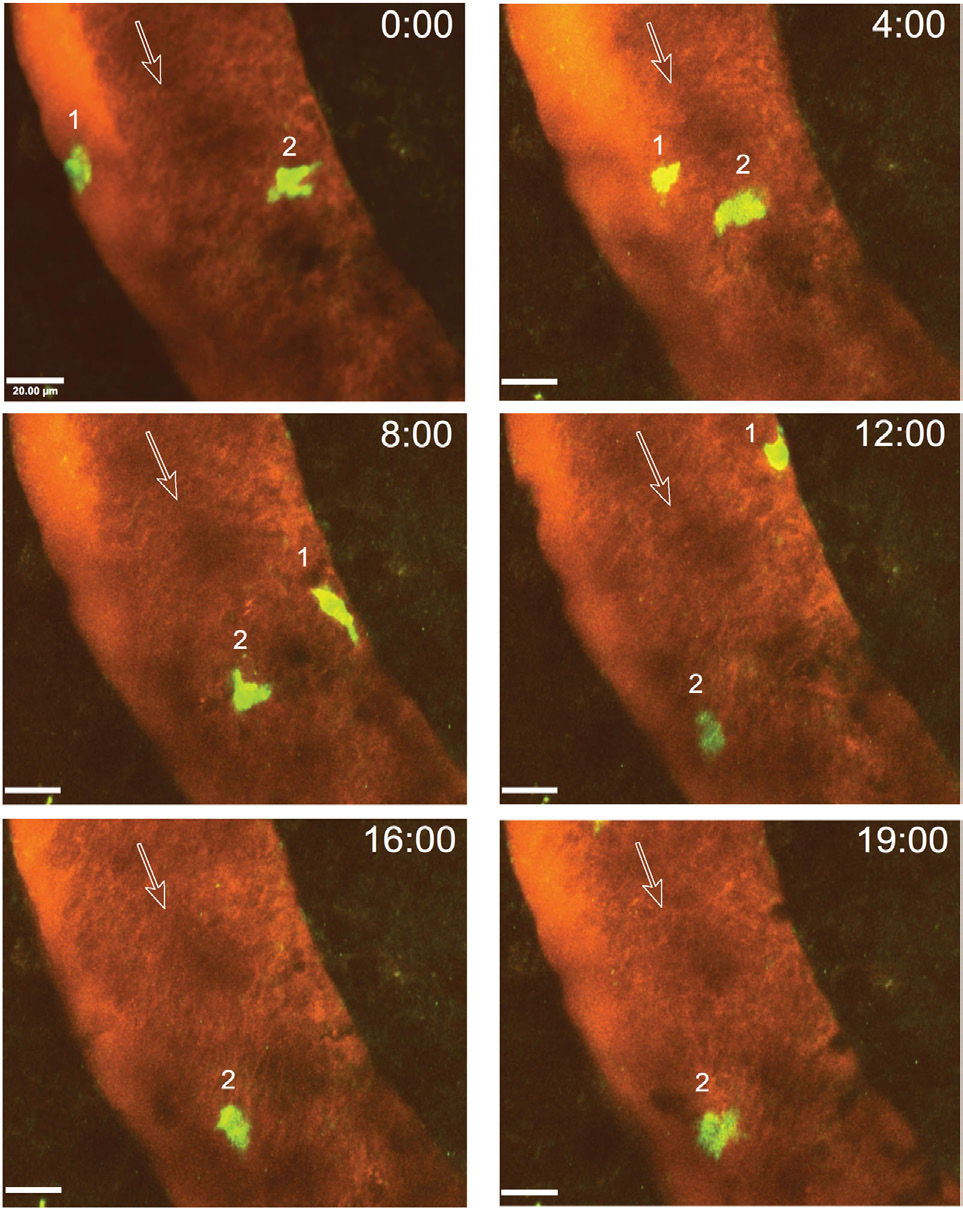
**Intraluminal crawling of activated 2D2 GFP CD4^+^ T cells within a cervical spinal cord post-capillary venule in a C57BL/6 mouse with experimental autoimmune encephalomyelitis (EAE)**. *In vitro* activated 2D2 GFP CD4^+^ T cells were systemically injected *via* the carotid artery catheter into a surgically prepared mouse with a clinical EAE score of 1 (hind leg weakness) at day 17 post-immunization. A *x*–*y*–*t* time-lapse sequence of a 150 µm × 150 μm scan field at a depth of 59–76 µm and 11 *z*-stacks with 1.7 µm spacing is shown. Contrast enhancement of the blood vessels was achieved by injection of Texas Red-dextran (MW = 70,000). Two CD4^+^ T cells are shown which crawled within the post-capillary venule. The numbers 1 and 2 indicate the two T cells visible in this sequence while the open arrows show the direction of blood flow. Movement of the crawling T cells is shown at 0, 4, 8, 12, 16, and 19 min of recording (Video S3 in Supplementary Material). T cell 1 crawled both with and against the direction of blood flow and left the regions of interest after 12 min. T cell 2 crawled only with the blood flow direction. GFP (green, CD4^+^ T cells) and Texas Red-dextran (red, blood plasma) were excited at 780 nm. Time is shown in minutes and seconds. Scale bar: 20 µm.

**Figure 7:**
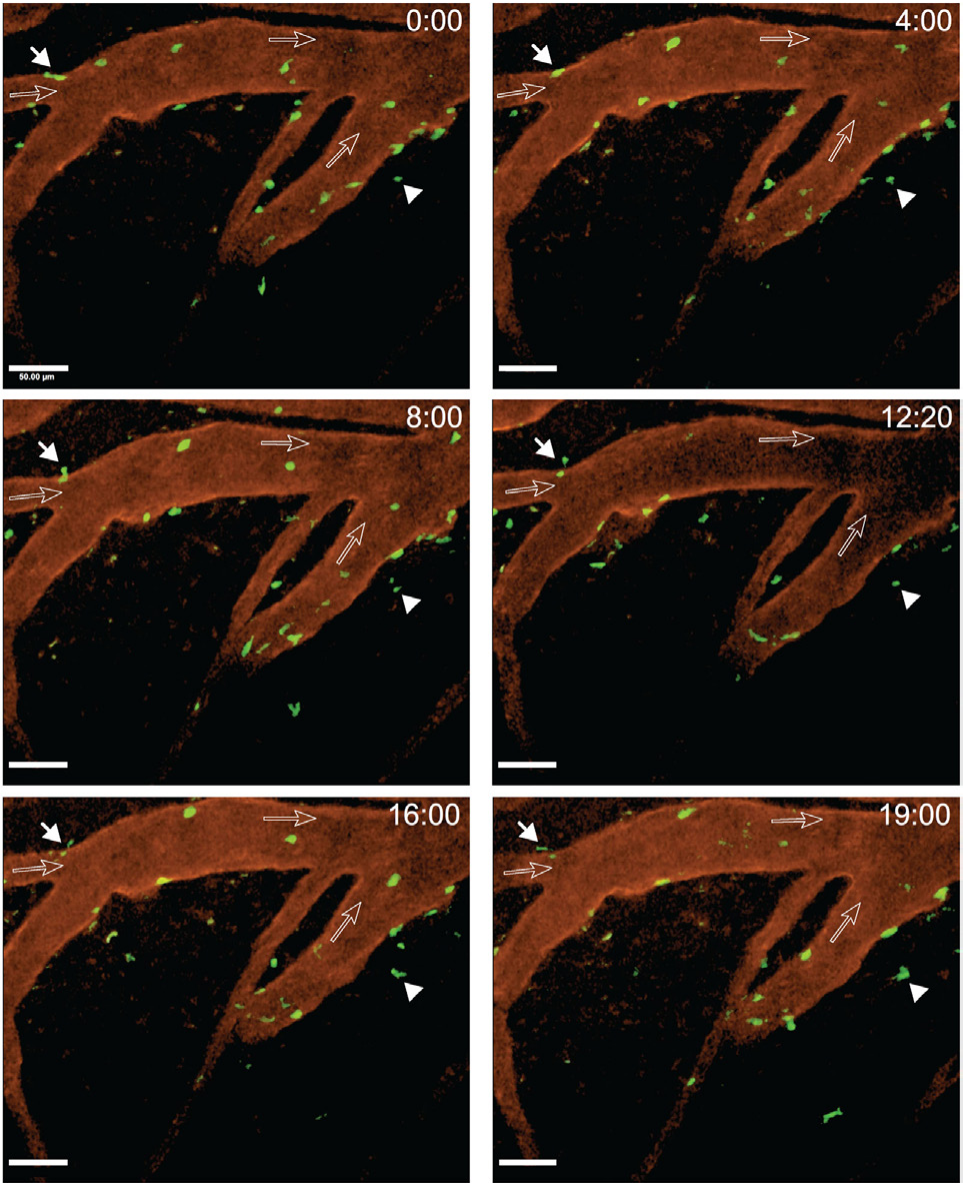
**Diapedesis of activated 2D2 GFP CD4^+^ T cells across cervical spinal cord post-capillary venules in C57BL/6 mice with experimental autoimmune encephalomyelitis (EAE)**. Laminectomy was performed on an EAE mouse showing onset of disease on day 22 post-immunization. Interactions of transferred *in vitro* activated 2D2 GFP CD4^+^ T cells with the post-capillary venules were monitored. A *x*–*y*–*t* time-lapse sequence of a 400 µm × 400 μm scan field at a depth of 47–91 µm and 12 *z*-stacks with 4 µm spacing is shown. Blood vessels were labeled with Alexa Fluor 594 conjugated anti-endoglin antibody. The filled arrow depicts a CD4^+^ T cell undergoing diapedesis visible from 8 to 19 min of recording. The filled arrowhead points out a CD4^+^ T cell moving outside of the vasculature. The open arrows show the direction of blood flow. Diapedesis of T cells was monitored at defined time points of 0, 4, 8, 12:20, 16, and 19 min of imaging (Video S6 in Supplementary Material). GFP (green, CD4^+^ T cells) and anti-endoglin (red, blood vessels) were excited at 780 nm. Time is shown in minutes and seconds. Scale bar: 50 µm.

After arrest on the vascular wall, we observed that CD4^+^ T cells started to crawl both with and against the direction of blood flow in cervical spinal cord post-capillary venules (Figure 6; Videos S3–S5 in Supplementary Material). Video 3 shows two CD4^+^ T cells over an observation time of 20 min. One of the T cells crawled with the direction of blood flow (cell number 2) and the other one crawled against the direction of blood flow (cell number 1) (Figure 6; Video S3 in Supplementary Material). T cell crawling could be observed as long as 20 min in the absence (Video S4 in Supplementary Material) or presence (Video S6 in Supplementary Material) of circulating fluorescently labeled T cells. Diapedesis of CD4^+^ T cells across the endothelial cell barrier of the post-capillary venules was also observed (Figure 7; Videos S6 and S7 in Supplementary Material). In Video S6 in Supplementary Material, we observed a total of 29 CD4^+^ T cells, 3 of these T cells could be directly observed to undergo diapedesis within the observation time of 19 min and 40 s (19:40). In another mouse, we observed a total of 15 CD4^+^ T cells interacting with the post-capillary venules, and 2 cells undergoing diapedesis were seen within a 15 min observation time (Video S7 in Supplementary Material).

**Figure 8:**
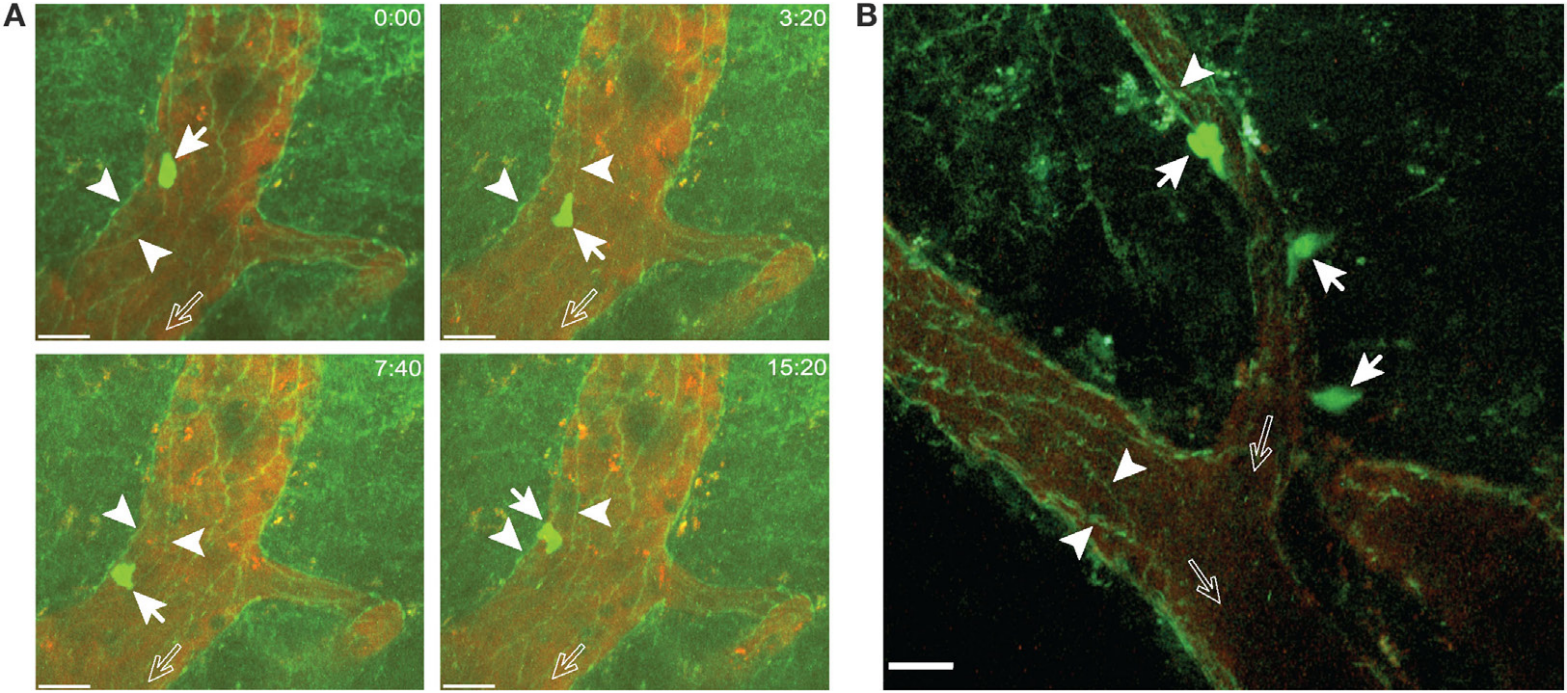
**Visualizing junctions of the blood–brain barrier endothelium in VE-cadherin-GFP knock-in mice with experimental autoimmune encephalomyelitis (EAE)**. *In vitro* activated 2D2 GFP CD4^+^ T cells were infused *via* the carotid catheter into VE-cadherin-GFP knock-in mice suffering from EAE at onset of disease. **(A)** A 2D2 CD4^+^ T cell (filled arrows) can be observed to crawl along the vascular junctions visualized by GFP (arrowheads) in a post-capillary venule of the cervical spinal cord. The open arrows show the direction of blood flow. Crawling of the T cell along the vascular junctions is shown at defined time points of 0, 3:20, 7:40, and 15:20 min of recording. Images were acquired from an area 200 µm × 200 µm at a depth of 135–145 µm and 11 *z*-stacks with 1 µm spacing. Contrast enhancement of the blood vessels was achieved by injection of Texas Red-dextran (MW = 70,000). GFP (green, CD4^+^ T cells and vascular junctions) and Texas Red (red, blood vessels) were excited at 800 nm. Time is shown in minutes and seconds. Scale bar: 20 µm. **(B)** 2D2 CD4^+^ T cells (filled arrows) were observed undergoing diapedesis across the wall of the post-capillary venule. Vascular junctions (arrowheads) in post-capillary venules of the cervical spinal cord are visible due to the GFP reporter. The open arrows show the direction of blood flow. Images were acquired from an area 200 µm × 200 µm at a depth of 40–60 µm and 11 *z*-stacks with 2 µm spacing. Blood vessels were labeled by injection of Alexa Fluor 594 conjugated anti-endoglin antibody. GFP (green, CD4^+^ T cells and vascular junctions) and anti-endoglin (red, blood vessels) were excited at 800 nm. Scale bar: 20 µm.

As outlined above, T cell diapedesis across the BBB can occur *via* a paracellular or a transcellular pathway as shown by live cell imaging of T cell migration across an *in vitro* model of the BBB derived from VE-cadherin-GFP knock-in mice (39, 49). VE-cadherin is a transmembrane protein of endothelial adherens junctions, thus the VE-cadherin-GFP fusion protein serves as a reporter for endothelial adherens junctions (40). As explained in Section “Methods,” EAE was induced in VE-cadherin-GFP knock-in C57BL/6 mice and a cervical spinal cord window was prepared for 2P-IVM exactly as outlined above in Section “Procedures of Mouse Preparation for Anesthesia, Cervical Spinal Cord Window Surgery, and Image Acquisition.” *In vitro* activated 2D2 GFP CD4^+^ T cells were systemically injected *via* a carotid artery catheter exactly as described in Section “Procedures of Mouse Preparation for Anesthesia, Cervical Spinal Cord Window Surgery, and Image Acquisition”. 2P-IVM allowed for visualization of the endothelial adherens junctions in post-capillary venules of VE-cadherin-GFP mice as green-fluorescent lines in the vascular walls *in vivo* (Figures 8A,B). GFP-2D2 T cells were observed to specifically crawl along the outline of these GFP-positive adherens junctions. Therefore, the VE-cadherin-GFP knock-in mice promise to be a valid model to address the cellular pathway of T-cell diapedesis across the BBB *in vivo* by using 2P-IVM imaging.

## Outlook

The described differences in the angioarchitecture of the lumbar versus cervical spinal cord blood vessels translate into differences in the hemodynamic parameters of cervical versus lumbar spinal cord post-capillary venules suggests differences in immune cell extravasation in these vascular beds. 2P-IVM imaging of immune cell extravasation across the cervical spinal cord microvasculature will thus provide novel insight into T-cell trafficking into the cervical spinal cord. In light of the importance of this spinal cord region in MS pathogenesis, imaging neuroinflammation in the cervical spinal cord is of utter importance. Finally, employing VE-cadherin-GFP knock-in mice might provide a novel and useful tool to image the cellular pathway of T-cell diapedesis across the BBB *in vivo* allowing to explore the neuroinflammatory conditions favoring the respective diapedesis pathways and opening up novel therapeutic targets for inhibiting immune cell entry into the CNS during neuroinflammation.

## Ethics Statement

Animal procedures were performed in accordance with the Swiss legislation on the protection of animals and were approved by the Veterinary Office of the Kanton of Bern (permission numbers: BE 42/14, BE 95/14, and BE 72/15).

## Author Contributions

NHJ performed the experiments including imaging, data analysis, and wrote the manuscript. HT performed the experiments and surgeries. GE contributed in the imaging setup and revising the manuscript. UD contributed in providing the mouse lines. NK, SB, and FZ contributed in providing the technology transfer and revising the manuscript. DV contributed in providing a mouse line. JS contributed in imaging acquisition, analysis of data, and revising the manuscript. BE supervised the project and finalized the manuscript.

## Conflict of Interest Statement

The authors declare that the research was conducted in the absence of any commercial or financial relationships that could be construed as a potential conflict of interest.

## Footnotes

^1^http://www.drzeintl.de/capde.htm.

^2^http://pubchem.ncbi.nlm.nih.gov/compound/3345#section=Top.

^3^http://pubchem.ncbi.nlm.nih.gov/compound/4192#section=Top.

^4^http://pubchem.ncbi.nlm.nih.gov/compound/68602#section=Top.

^5^https://www.thermofisher.com/order/catalog/product/Q21021MP.

## Supplementary Material

Video S1 **Arrest of activated 2D2 GFP CD4^+^ T cells within inflamed cervical spinal cord post-capillary venules during experimental autoimmune encephalomyelitis (EAE)**. Active EAE was induced as described in Section “Methods.” Laminectomy was performed (described in step 15 of the procedures) on day 14 post-immunization when the mouse showed onset of disease. *In vitro* activated CD4^+^ T cells from “2D2-GFP”-mice were injected *via* a carotid catheter before 2P-IVM imaging. Blood vessels were labeled by injection of Alexa Fluor 594 conjugated anti-endoglin antibody. GFP (green, CD4^+^ T cells) and anti-endoglin (red, blood vessels) were excited at 800 nm. A *x*–*y*–*t* time-lapse sequence of a 400 μm × 400 μm scan field at a depth of 59–91 µm and 9 *z*-stacks with 4 μm spacing shows the arrest of CD4^+^ T cells within post-capillary venules of the cervical spinal cord. Time is shown in minutes and seconds. Scale bar: 50 μm. This video is the source video of which images shown in Figure 5 were taken.

Video S2 **Arrest of activated 2D2 GFP CD4^+^ T cells within inflamed cervical spinal cord post-capillary venules during experimental autoimmune encephalomyelitis (EAE)**. Active EAE was induced as described in Section “Methods.” Laminectomy was performed (described in step 15 of the procedures) on day 22 post-immunization when the mouse showed onset of disease. *In vitro* activated CD4^+^ T cells from “2D2-GFP”-mice were injected *via* a carotid catheter before 2P-IVM imaging. Blood vessels were labeled by injection of Alexa Fluor 594 conjugated anti-endoglin antibody. GFP (green, CD4^+^ T cells) and anti-endoglin (red, blood vessels) were excited at 780 nm. A *x*–*y*–*t* time-lapse sequence of a 400 μm × 400 μm scan field at a depth of 47–91 μm and 12 *z*-stacks with 4 µm spacing shows the arrest of CD4^+^ T cells within post-capillary venules of the cervical spinal cord. Firmly arrested T cells are seen to neither move nor detach from the vessel wall within a timeframe of 20 s. Time is shown in minutes and seconds. Scale bar: 50 μm.

Video S3 **Intraluminal crawling of activated 2D2 GFP CD4^+^ T cells in inflamed cervical spinal cord post-capillary venules during experimental autoimmune encephalomyelitis (EAE)**. Active EAE was induced as described in Section “Methods.” Laminectomy was performed (described in step 15 of the procedures) on day 17 post-immunization when the mouse showed the clinical score of 1 (hind leg weakness). *In vitro* activated CD4^+^ T cells from “2D2-GFP”-mice were injected *via* a carotid catheter before 2P-IVM imaging. Contrast enhancement of blood vessels was achieved by injection of Texas Red-dextran (MW = 70,000). GFP (green, CD4^+^ T cells) and Texas Red were excited at 780 nm. A *x*–*y*–*t* time-lapse sequence of a 150 μm × 150 μm scan field at a depth of 59–76 μm and 11 *z*-stacks with 1.7 μm spacing is shown. Two CD4^+^ T cells are seen, which crawl within the post-capillary venule. Time is shown in minutes and seconds. Scale bar: 20 µm. This video is the source video of which images shown in Figure 6 were taken.

Video S4 **Intraluminal crawling of an activated 2D2 CD4^+^ T cell in inflamed cervical spinal cord post-capillary venules during experimental autoimmune encephalomyelitis (EAE)**. *In vitro* activated 2D2 CD4^+^ T-cells labeled with fluorescent CellTracker CMAC were systemically injected *via* the carotid artery catheter into a surgically prepared mouse at the onset of EAE. In the regions of interest, one transferred CD4^+^ T cell was observed to crawl along the direction of blood flow until 4 min and 20 s of recording. At this time, the T cell changed the direction of crawling against the blood flow until minute 12 of the recording. At this time point, the observed T cell again changed the direction of crawling along the direction of blood flow and continued to crawl to the end of 20 min of recording. A *x*–*y*–*t* time-lapse sequence of a 200 μm × 200 μm scan field at a depth of 79–100 μm and 8 *z*-stacks with 3 µm spacing is shown. Blood vessels were labeled by injection of Alexa Fluor 594 conjugated anti-endoglin antibody. CMAC (blue, CD4^+^ T cell) and anti-endoglin (red, blood vessels) were excited at 780 nm. Time is shown in minutes and seconds. Scale bar: 20 μm.

Video S5 **Intraluminal crawling of activated 2D2 CD4^+^ T cells in inflamed cervical spinal cord post-capillary venules during experimental autoimmune encephalomyelitis (EAE)**. *In vitro* activated 2D2 GFP CD4^+^ T cells and 2D2 CD4^+^ T cells labeled with fluorescent CellTracker CMAC were systemically injected *via* the carotid artery catheter into a surgically prepared EAE mouse at the onset of disease. A CMAC labeled T cell (blue cell) is seen to crawl against the direction of blood flow for the entire 20 min of recording. A GFP^+^ T cell is seen to crawl against the direction of blood flow until 3 min of the recording when it detached and re-entered circulation. Another GFP^+^ T cell (at time point of 13 min) and two additional CMAC labeled T cells (at time points of 8 min:40 s and 9 min:40 s) can be observed to transiently arrest on and crawl along the vascular wall and to rapidly re-enter blood circulation. A *x*–*y*–*t* time-lapse sequence of a 300 μm × 300 μm scan field at a depth of 52–115 μm and 16 *z*-stacks with 4.2 μm spacing is shown. Blood vessels were labeled by injection of Alexa Fluor 594 conjugated anti-endoglin antibody. GFP (green, CD4^+^ T cells), CMAC (blue, fluorescently labeled CD4^+^ T cells), and anti-endoglin (red, blood vessels) were excited at 780 nm. Time is shown in minutes and seconds. Scale bar: 30 μm.

Video S6 **Diapedesis of activated 2D2 CD4^+^ T cells across inflamed cervical spinal cord post-capillary venules during experimental autoimmune encephalomyelitis (EAE)**. Active EAE was induced as described in Section “Methods.” Laminectomy was performed (described in step 15 of the procedures) on day 22 post-immunization when the mouse showed onset of disease. *In vitro* activated CD4^+^ T cells from “2D2-GFP”-mice and 2D2 CD4^+^ T cells labeled with fluorescent CellTracker CMAC were systemically injected *via* a carotid catheter before 2P-IVM imaging. A *x*–*y*–*t* time-lapse sequence of a 400 μm × 400 μm scan field at a depth of 47–91 μm and 12 *z*-stacks with 4 μm spacing is shown. Blood vessels were labeled by injection of Alexa Fluor 594 conjugated anti-endoglin antibody. CD4^+^ T cells undergoing diapedesis and crawling outside of the vasculature can be observed during 20 min of recording. Due to the unavoidable limited movement of the mouse, the image sequences were cropped at 05:40, 07:00, and 11:20 min. GFP (green, CD4^+^ T cells), CMAC (blue, fluorescently labeled CD4^+^ T cell), and Alexa Fluor 594 (red, blood vessels) were excited at 780 nm. Time is shown in minutes and seconds. Scale bar: 50 μm. This video is the source video of which images shown in Figure 7 were taken.

Video S7 **Diapedesis of activated 2D2 GFP CD4^+^ T cells across inflamed cervical spinal cord post-capillary venules during experimental autoimmune encephalomyelitis (EAE)**. Laminectomy (described in step 15 of the procedures) was performed in a C57BL/6 mouse at the onset of EAE. *In vitro* activated CD4^+^ T cells from “2D2-GFP”-mice were systemically injected *via* a carotid catheter before 2P-IVM imaging. During the recording of 15 min, two CD4^+^ T cells undergoing diapedesis across cervical spinal cord post-capillary venules can be observed. A *x*–*y*–*t* time-lapse sequence of a 300 μm × 300 μm at a depth of 60–112 μm and 14 *z*-stacks with 4 µm spacing is shown. Blood vessels were labeled with Alexa Fluor 594 conjugated anti-endoglin antibody. GFP (green, CD4^+^ T cells) and Alexa Fluor 594 (red, blood vessels) were excited at 780 nm. Time is shown in minutes and seconds. Scale bar: 40 µm.
